# COVID-19 pandemic is associated with increased sleep disturbances and mental health symptoms but not help-seeking: a cross-sectional nation-wide study

**DOI:** 10.5935/1984-0063.20220027

**Published:** 2022

**Authors:** Claudia Roberta de Castro Moreno, Silvia G. Conway, Márcia Assis, Pedro Rodrigues Genta, Daniela V. Pachito, Almir Tavares Jr, Danilo A. Sguillar, Gustavo Moreira, Luciano F. Drager, Andrea Bacelar

**Affiliations:** 1 Brazilian Sleep Association, São Paulo, Brazil.; 2 School of Public Health, University of São Paulo, Brazil.; 3 Stress Research Institute, Department of Psychology, Stockholm University, Sweden.; 4 Akasa - Formação e Conhecimento, São Paulo, Brazil.; 5 Psychiatry Department, University of São Paulo Medical School, São Paulo, Brazil.; 6 Clínica do Sono de Curitiba, Hospital São Lucas, Curitiba Paraná, Brazil.; 7 Fundação Getúlio Vargas, Brazil.; 8 Compromisso Social, Hospital Sírio-Libanês, São Paulo, Brazil.; 9 Neurosciences Program and Department of Mental Health, Federal University of Minas Gerais, Brazil.; 10 ENT Department of the Federal University of São Paulo, Brazil.; 11 Department of Pediatrics and Psychobiology, Universidade Federal de São Paulo, Brazil.; 12 Laboratório do Sono, LIM 63, Pulmonary Division, Heart Institute (InCor), Hospital das Clínicas HCFMUSP, Universidade de São Paulo, São Paulo, SP, BR.; 13 Unidade de Hipertensão, Instituto do Coração (InCor), Hospital das Clinicas HCFMUSP, Faculdade de Medicina, Universidade de São Paulo, São Paulo, Brazil.; 14 Carlos Bacelar Clinica, Rio de Janeiro, Brazil.

**Keywords:** Sleep, COVID-19, Help-Seeking Behavior, Mental Health

## Abstract

**Objective:**

This study aimed firstly to describe sleep-related and mental health symptoms before and during the COVID-19 pandemic in a national-wide sample and, secondly, to verify attitudes towards help-seeking to treat these symptoms.

**Material and Methods:**

Data were collected through an online questionnaire sent through the Brazilian Sleep Association’s social media. The questionnaire included sociodemographic and sleep aspects questions currently and before the pandemic period. In addition, the survey addressed current and previous anxiety, depression, and burnout symptoms. The outcome help-seeking was addressed in the questionnaire as well by a single question asked when the participant reported mental or sleep problems.

**Results:**

The study covered 6,360 participants, mean age 43.5 years (SD=14.3), 76.7% female and 63.7% with undergraduate or higher degree filled out the survey. Seventy percent of participants reported sleep disturbances and 80% reported symptoms of anxiety during the pandemic. Help-seeking behavior was found only in one third of them. Hours of sleep reduced from 7.12 to 6.2h, which can be related with the increase in 28.2% of dissatisfaction with sleep duration during the pandemic. The highest frequency of complaints related to sleep was difficulty to fall asleep three or more times a week (going from 27.6% before the pandemic to 58.9% during the pandemic; p<0.001). Moreover, it was observed that help-seeking was more prevalent in men than women, and more in younger participants than in older ones.

**Conclusion:**

There was an increase of sleep and mental self-reported problems during the pandemic, which was not followed by help-seeking.

## INTRODUCTION

Currently, there are hundreds of published studies about COVID-19, the 21^st^ century pandemic. Many of them have discussed aspects related to SARS-CoV-2 virus^1,2^, the etiologic agent of COVID-19, while others have investigated vaccines’ efficacy^3,4^ or long-term effects of the disease^5,6^.

There are also several studies exploring how society dealt with the pandemic. Most of them are related to the consequences of lockdown or the pandemic restrictions as the stay-at-home recommendations^7-11^. Yet, individuals exposed to the disease or infected by the virus during the pandemic were subjected to quarantine or isolation, respectively. A study of the latter has shown that they were at increased risk of adverse mental health outcomes, mainly depression, anxiety and stress-related disorders and anger when compared to the uninfected or non-diseased population^12^. Considering the very well established association between mental health and sleep, it is not surprising that sleep problems have raised since the pandemic has started^13,14^. Several studies investigated sleep problems and evaluated whether and how sleep quality was affected during the 2020-2021 period in several countries^15-19^. These studies identifying effects either of the pandemic restrictions (e.g., stay-at-home orders) or the isolation or quarantine on sleep schedules/sleep quality in children, adolescents, adults, and patients with comorbidities^20-24^.

The COVID-19 pandemic led to the development of new episodes of insomnia or exacerbated pre-existing insomnia in part of the population, as those who remained working in-person, such as healthcare professionals^25-27^. As demonstrated by our group through a survey with 4,364 healthcare professionals, sleep quality worsened among 61.4% of the participants throughout the pandemic. In addition, 43.5% reported ≥1-hour sleep duration reduction and, insomnia was prevalent in 1,444 (32.9%) participants^25^.

In general, insomnia has negative impact and is strongly associated to mental disorders^14^. Despite being a treatable and potentially preventable disorder, insomnia remains an under-recognized, under-diagnosed, and under-treated condition^14,28^. Help-seeking seems to be postponed and, in some cases absent, aggravating burden and costs. Insomnia untreated is more likely to disrupt labor, social and family functioning, harming the quality of life^29^ and is associated with risk for psychiatric, metabolic and cardiovascular diseases^14,30,31^. Barriers to help-seeking behavior involving sleep disorders in children and adults, including insomnia and mental illness, have been focused by some studies on the past decade^32-38^.

Our hypothesis is that help-seeking was reduced during the pandemic, with less people seeking professional help to treat sleep or mental problems. In this context, this study aimed firstly to describe sleep-related and mental disorders symptoms before and during the COVID-19 pandemic in a national-wide sample and, secondly, to verify attitudes towards help-seeking to treat these symptoms.

## MATERIAL AND METHODS

This was a cross-sectional study carried out by the School of Public Health, University of São Paulo, in collaboration with the Brazilian Sleep Association. Data were collected through an online questionnaire. The invitation to participate was sent through the Brazilian Sleep Association’s social media. Data collection was carried out from November 2020 to April 2021. The study was approved by the Research Ethics Committee of the Faculty of Public Health of the University of São Paulo (Process No. #39395520.8.0000.5421). Participants were informed of the purpose of the study and consented to the use of their data. The power of the sample was 95%, which was calculated using G*Power software, with the repeated measures test (pre and pandemic) as reference.

The questionnaire was developed by a joint initiative effort from sleep researchers of the Brazilian Sleep Association and the Brazilian Medical Sleep Association. The participants were invited to answer questions about sleep quality, sleep duration, and current and pre-pandemic sleep symptoms. Sociodemographic data regarding age, sex, educational level, and occupation were included in the questionnaire. Lifestyle habits as alcohol consumption and its frequency were included as well. Nutritional status information was used to calculate body-mass index (BMI). The nutritional status was characterized according to the WHO classification.

Sleep aspects included difficulty in falling asleep, difficulty to maintain sleep, and waking up early in the morning and not going back to sleep. Sleep hours was an open question, and a Likert scale was used to assess satisfaction with sleep duration. Moreover, the survey also included questions about the occurrence of nightmares, snoring, sleep apnea, bruxism, restless legs symptoms, somnambulism, and naps before and during the pandemic period. In addition, the participants were asked to declare the frequency of all the sleep problems before the pandemic and currently.

Regarding mental health, the survey included questions addressing current and previous anxiety, depression, and burnout symptoms. Questions related to anxiety symptoms included feeling nervous, anxious or not being able to stop or controlling worrying. These anxiety symptoms were approach through the Generalized Anxiety Disorder Scale-2 (GAD-2)^39^. The questionnaire also included the frequency of depressed mood and anhedonia by addressing little interest or pleasure in doing things and feeling down, depressed or hopeless. The frequencies of both symptoms were classified in “not at all, several days, more than half of the days, nearly every day”^40^. In addition, the questionnaire also included a single question addressing current or previous burnout symptoms^41^. The outcome help-seeking was addressed in the questionnaire as well, with a single question asked when the participant reported mental or sleep problems.

### Statistical analyses

Descriptive analysis was performed using means, medians, standard deviations, minimum and maximum values for quantitative variables and absolute frequencies and proportions for qualitative variables. Statistical tests were performed to compare the results of variables of interest according to the evaluation period (before and during the coronavirus pandemic). Shapiro test was performed to verify the adherence of the variables to the normal distribution. As the quantitative variables showed a nonparametric distribution, the Wilcoxon test was used to compare repeated measures for dichotomous variables. To compare categorical repeated variables, the McNemar test for dichotomous variables and the McNemar-Bowker test for variables with 3 or more categories were used. Spearman’s rank correlation coefficient was performed to check the correlation between ages and sleep duration. A cutoff of 5 hours of sleep was defined to compare hours of sleep and perception of insufficient sleep reported.

Filling errors were considered missing data, which explains the small variation in sample size in some analyses. To perform the statistical analysis, STATA software version 14 and Statistica version 12 were used.

## RESULTS

Among the 6,360 participants, the highest frequencies of participation were observed in São Paulo (29.9%) and Rio de Janeiro (22.2%), corresponding to 52.2% of the sample. When observing the geographic regions, the highest proportions occurred in the Southeast (59.7%), Northeast (16.6%) and South (14.7 %). Participants aged 30 to 59 years comprised the majority of the sample (66.5%), with average age 43.5 years old (SD=14.3), ranging from 10 to 87 years old. Most participants were female (76.7%) (Table 1). As for having children, 57.3% answered affirmatively, and among these, the number of children ranged from 1 to 12, with 81% having 1 or 2 children (mean=1.9; SD=0.9 children). Regarding education, the largest proportions of participants had graduate degrees (35.7%) or completed higher education. As for the nutritional status, assessed through the BMI, the highest proportions were people with adequate weight (37.9%), followed by overweight (33.1%), and obese (25%). The mean BMI was 27.0kg/m^2^ (SD=5.6), ranging from 16 to 71.3kg/m^2^, with a median of 26.1kg/m^2^. Alcohol consumption went from 7% before the pandemic to 9.2% during the pandemic among the participants.

**Table 1:** Distribution of the study population according to sociodemographic data.

Variable	n	%
**Age group**		
10 to 19	23	3.7
20 to 49	386	60.8
50 to 59	129	20.4
60 +	961	15.1
**Gender**		
Female	4877	76.7
Male	1474	23.2
Other	9	0.1
**Children**		
No	2716	42.7
Yes	3644	57.3
**Education**		
Elementary school	355	5.5
High school	1956	30.8
Undergraduate degree	1780	28.0
Graduate degree	2269	35.7
**Occupational status**		
Retired	785	12.3
Unemployed	1002	15.8
Employee	2286	35.9
Entrepreneur/employer	376	5.9
Student	644	10.1
Self-employed	1267	19.9
**Nutritional status (Body Mass Index)**		
Underweight	206	3.2
Eutrophic	2413	37.9
Overweight	2108	33.1
Obesity	1592	25.0
Not informed	41	0.6
**Total**	**6,360**	**100**

**Figure 1:**
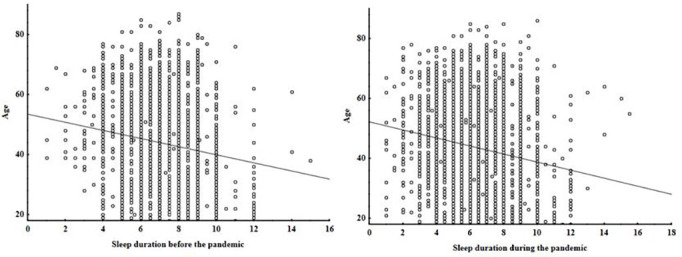
Age and sleep duration before and during the pandemic of participants over 18 years old. **A.** Before the pandemic (r=-0.13; *p*<0.05); **B.** During the pandemic (r=-0.17; *p*<0.05); n=6,360. Spearman correlation significant at *p*<0.05.

There was a statistically significant decrease in the number of hours of sleep from the period before (mean=7.12 hours; SD=1.34 hours) the pandemic to during the pandemic (mean=6.23 hours; SD=1.72 hours) (*p*<0.001). In order to check an ageing effect on sleep duration, we ran a correlation analysis between the ages of participants and sleep duration. As expected, there was a negative correlation (although weak) between sleep duration and age, which can be observed before the pandemic (r=-0.13; *p*<0.05) and during the pandemic (r=-0.17; *p*<0.05) (Figures 1A and 1B).

When sleep duration was dichotomized into more than 5 hours per day and less than 5 hours per day, it was observed that there were changes (p<0.001) in the frequency of people reporting insufficient sleep: 211 (3.4%) participants before the pandemic and 1,042 (16.7%) participants during the pandemic.

There was a statistically significant increase (*p*<0.001) in the frequency of dissatisfaction with sleep duration when comparing the period before the pandemic (44.5%) with the period during the pandemic (72.75%). When comparing the two periods, there was also a statistically significant worsening (*p*<0.001) in sleep quality, with the frequency of participants reporting poor quality rising from 21% to 53.4% (Table 2).

**Table 2:** Distribution of the study population according to sleep quality and satisfaction with sleep duration.

Variable	Before the pandemic		During the pandemic		p
	n	%	n	%	
**Satisfaction with sleep duration**					
Satisfaction	3,530	55.5	1,738	27.3	<0.001*
No satisfaction	2,830	44.5	4,622	72.7	
**Sleep quality**					<0.001**
Good	2,456	38.6	1,152	18.1	
Regular	2,497	39.3	1,735	27.3	
Bad	1,335	21.0	3,397	53.4	
I do not know	72	1.1	76	1.2	
**Total**	**6,356**	**100.0**	**6,360**	**100.0**	

* McNemar test for repeated measures;

** McNemar-Bowker test for repeated measures.

**Figure 2:**
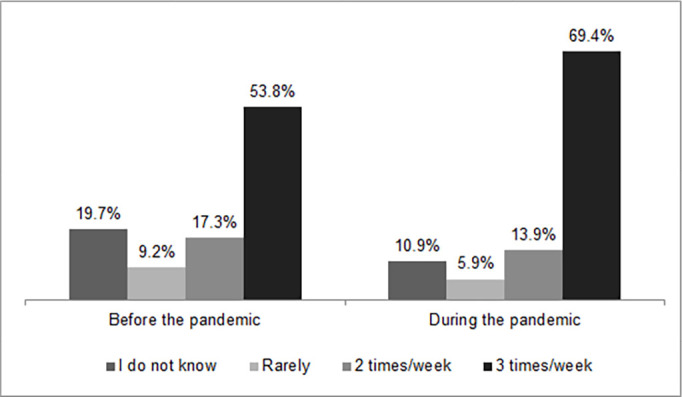
Distribution of the study population according to the frequency of sleep problems reported.

The high percentage of participants reporting poor sleep quality is corroborated by the percentage of people who reported having sleep problems 3 or more times a week, which went from 53.8% before the pandemic to 69.4% in the pandemic period (Figure 2).

Left = before the pandemic; Right = during the pandemic; n=6,360.

During the pandemic, the highest frequencies of complaints related to sleep were difficulty to fall asleep three or more times a week. Furthermore, it was also observed that there were changes characterized by increase in frequency in this category from before the pandemic to during the pandemic (*p*<0.001), going from 27.6% to 58.9% (Table 3). Moreover, the difficulty in falling asleep more frequently (three or more times a week) was more prevalent among those participants with no higher education level (68.2%) when compared to those with higher education (53.5%) (*p*<0.05), representing 59.9% of all participants. There was increase in the frequency of those who sometimes wake up early without going back to sleep from 33.6% to 35.7%, and 15.5% reported that it always happened before the pandemic but went to 35.5 % currently. The frequency of participants who took naps also increased from before compared to during the pandemic (*p*<0.001), going from 30.7% to 40.7%. Waking up too early and not going back to sleep also changed from before to during the pandemic (*p*<0.001).

**Table 3:** Distribution of the study population according to difficulty in falling asleep, before and during the pandemic.

Variable	Before the pandemic		During the pandemic		p
	n	%	n	%	
None	2,161	34.0	1,355	21.3	<0.001
Once a week/less than twice a week	2,440	38.4	1,262	19.8	
Three or more times a week	1,755	27.6	3.743	58.9	
**Total**	**6,356**	**100.0**	**6,360**	**100.0**	

**Figure 3:**
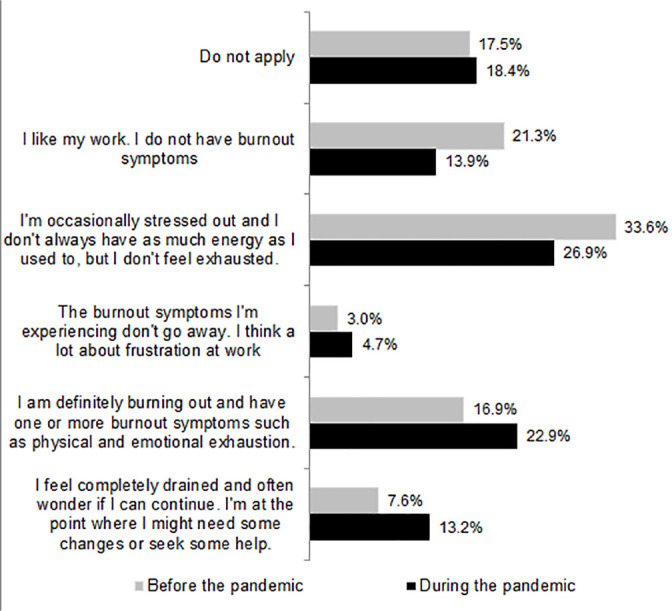
Presence of burnout symptoms among the total number of participants and period.

Among participants, 80% reported increased symptoms of anxiety. About half of the participants (50.1%) reported little interest/pleasure in doing things and/or feeling sad/depressed/lack of hope, and in 20.8% this feeling occurred in more than half of the days and in 29.3% occurred almost every day. The feeling of nervousness/anxiety and/or inability to control worries was present for 58.1% of the participants, and in 26%, this feeling occurred in more than half of the days and in 32.1% it occurred almost every day. It was observed that there was increase in the symptoms indicative of burnout (Figure 3).

Gray = before the pandemic; Black = during the pandemic; n=6,360.

Symptoms related to burnout were grouped in order to characterize the presence of this syndrome. It was observed that there were changes (*p*<0.001) in the frequency of burnout complaints from the period before the pandemic to during the pandemic, with an increase from 27.5% to 40.8%.

**Figure 4:**
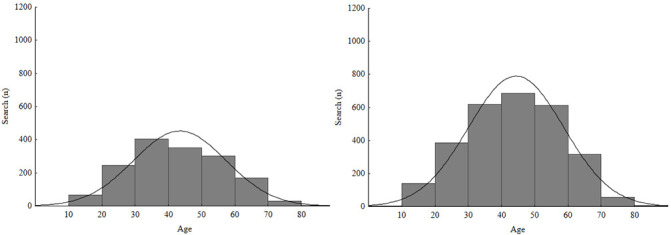
Distribution of participants who reported sleep problems more than three times a week, according to help-seeking and age.

Among the total number of participants, 33% reported help-seeking. It is interesting to observe that participants of all ages sought medical help. Yet, among the participants with sleep problems more than three times a week, those who did not seek professional help were older than those who sought for help (*p*=0.014). Out of 4,394 who reported sleep problems more than three times a week only 1,572 sought help (Figure 4). In addition, out of 79% women who reported sleep problems 77.3% did not seek professional help. Among men, only 22.5% out of 20.8% who reported sleeping problems did not seek help.

Left = sought help (n=1,572); Right = did not help-seek (n=2,822).

When those who reported some type of mental health problem during the pandemic were selected (5,370 people; 84.4% of participants), 36.1% sought professional help.

## DISCUSSION

To our knowledge, this is the first Brazilian nationwide study that investigated help-seeking attitudes for sleep and mental symptoms during the COVID-19 pandemic. We found that about 70% of participants reported sleep problems three or more times a week, 80% reported increase in some kind of mental health symptoms. In addition, 50% reported anhedonia-related symptoms (or depressive-related symptoms). It was observed that the frequency of all symptoms increased from pre to during the pandemic period. The three sleep problems reported more than three times a week (difficulty to fall asleep, sometimes waking up early without going back to sleep, waking up too early and not going back to sleep) demonstrated that insomnia symptoms were the most reported sleep problem, which increased by 32% during the pandemic. These findings were observed mainly among people with lower education level. Nevertheless, help-seeking attitudes were observed only in 35.7% of the sample, more in men than women, more in younger persons than in older ones.

In a study focused on mental health symptoms during the pandemic, the authors found that COVID-19 concerns had a positive and direct effect on attitudes toward seeking professional help, mediated by anxiety scores^42^. On the other hand, higher immunity and exaggerated perception had negative effects on attitudes toward seeking psychological health. Nevertheless, according to a group of researchers in UK, the recognized cutoff point on standardized instruments may not be ideal for the current situation^43^. Besides, these instruments are too long to be used routinely during the pandemic, and the lack of a good and easy-to-apply instrument maybe be among the reasons for not searching medical help. In other words, the authors argue that people are not aware about their mental symptoms.

What draws the attention in our study is that almost 70% of the participants reported sleep problems. Even though these problems are easier to perceive than mental disorder symptoms, only one third sought professional help (1,572 out of 4,394). Moreover, other findings during the pandemic points out to self-neglect attitudes toward self-care: reduction in sleep duration in more than 50 minutes; increase of 28% of participants dissatisfied with sleep duration and increase of 32% in those dissatisfied with sleep quality. A possible explanation for that relies on the increase of participants who took naps during the pandemic. As reported some years ago, people try various strategies to combat their sleep problems prior to the next step, which is seeking help^32,33^. This habit might be potentiated by the pandemic. Perhaps the participants believed that taking naps was an alternative to deal with depression, anxiety and insufficient sleep. However, even with the increase of naps, the number of participants sleeping less than five hours and dissatisfied with sleep duration soared during the pandemic. These results suggest that the naps were insufficient, possibly increasing the dissatisfaction with sleep quality and contributing to the vicious circle of insomnia. Considering that insomnia symptoms were more prevalent among people with low education level, the inhibited frequency of help-seeking behavior may reflect a lack of comprehension of sleep hygiene habits. There are also other possible hypotheses not investigated in this study as poor self-perception of mental conditions, unfamiliarity of risks related to sleep problems, fear of COVID infection or even lack of healthcare assistance accessibility. Henssler et al. (2021)^12^ have demonstrated that lower levels of education, social capital, and household income, including financial loss or economic impact in pandemics, were associated with more severe symptoms of stress-related disorders, unspecified psychological disorders, depression, anxiety, and poor sleep quality.

In a study that included cervical cancer patients, the major reasons for delayed seeking medical care was lack of awareness in identifying symptoms and patients assuming that the symptom would resolve by itself. The authors also found that the odds of patient delay were 3.2 times higher in the age group ≥60 years compared to the age group below 45 years^44^. We also observed in the present study that help-seeking attitudes are more prevalent among younger persons than older ones. A hypothesis to explain this result might be the high prevalence of COVID among elderly people, which could make this part of the population more afraid of getting out of home to seek for health assistance.

On the other hand, in an Australian study involving healthcare workers with symptoms of psychological problems, attitudes and help-seeking behaviors were more prevalent among the older ones^45^. Older healthcare workers had a more positive perspective and less prejudice, whereas younger ones were most likely to report barriers to seeking help. Nevertheless, when burnout was the matter, professionals from the healthcare system reported a stigma for help-seeking behavior^46^. Thus, it is not surprising that burnout symptoms and insomnia disorder were highly prevalent among healthcare workers in previous studies^25-27^.

In the present study, conducted from nine to 12 months after the stay-at-home orders, the prevalence of burnout increased by more than 13%, reaching more than 40% of the participants. Our results reinforce the need to face the barriers for help-seeking behavior. As demonstrated by other studies, help-seeking is a complex system, involving factors related to age, education level, health knowledge, perception of current health status, illness stigmatization, household income, social support and training and support among healthcare assistances^12,35-36,44^.

Particularly, the organization and structure of the healthcare assistance is another problem that demands attention. Training programs of healthcare personnel for assisting mental and sleep problems in the population could mitigate the resistance to help-seeking^47^. Several studies reinforced the need for a comprehensive approach that encompasses knowledge on sleep disorders diagnoses and therapeutics to general health professionals^47^.

Our study has some limitations. First, we have asked about health conditions before the pandemic, which depends on memory recall of the participants. It is also possible that those with sleep problems were more interested in participating. In addition, as in other studies, most participants were women, as they generally volunteer more for a survey than men do. Thus, we cannot disregard a selection bias. We did not distinguish healthcare professionals from the general population. On the other hand, our aim was to look at the population as a whole, covering all age groups regardless of professions. Moreover, although we did not plan to address each region of the country, therefore, the sample is not representative; we do have participants from all regions. Finally, we need to take into account that this study has a cross-sectional design that means we cannot establish cause-effect relationships between the studied variables.

In short, while comparing sleep and mental health symptoms before and during the COVID-19 pandemic, we found 70% of participants reporting sleep problems and 80% reporting symptoms of mental disorder during the pandemic. Help-seeking behavior was found only in one third of them. Moreover, help-seeking was more prevalent in men than women, and more in younger persons than in older ones. This study points out the need to develop outreach programs in sleep knowledge among the general population.
